# Supplementary material for: Patterns of Intron Gain and Loss in Fungi
Source: PLoS Biol. 2004 Nov 30;2(12):e422. doi: 10.1371/journal.pbio.0020422 (PMC532390; doi:10.1371/journal.pbio.0020422)
Supplement: Table S1 — Also available at http://genes.mit.edu/NielsenEtAl/. (4.3 MB ZIP). [file pbio.0020422.st001.zip › NielsenEtAl/html/649.html]

AN3417.1.NCU02107.1.MG04646.1.FG00287.1


```
 CLUSTAL W (1.82) Multiple Sequence Alignments - Introns Inserted


Sequence 1: NCU02107.1	577 aa
Sequence 2: MG04646.1	599 aa
Sequence 3: FG00287.1	595 aa
Sequence 4: AN3417.1	750 aa
Alignment Length: 788 aa
Number Identitical Residues: 273 aa
Alignment Score (without introns) 13610


MG04646.1 	------------------------------------------------------------
NCU02107.1	------------------------------------------------------------
FG00287.1 	------------------------------------------------------------
AN3417.1  	MASDREVVEAEKSGTFSLMRSSMGVADEDILGMVVEKRRRETRPRRTRPGIYILYEERKG
          	 ::. .  .:..:.: :   :: . :...  .   ..   .: .  : ..     .. ..

MG04646.1 	------------------------------------------------------------
NCU02107.1	------------------------------------------------------------
FG00287.1 	------------------------------------------------------------
AN3417.1  	ENGKEDCMVATGRGRFNAVLRVIRFDRERIGESFVALFGMVGSSPGSQVAFSKSRSFFVL
          	.......  ::. .  .:       . .  ..:  :  .  .::..:. : :.: :    

MG04646.1 	-----------------~------------MASS---TTLPKLPIFEAIRKHDPESTAVV
NCU02107.1	-----------------~------------MAS-----TLPKLPVFEAISRHNPESTVVV
FG00287.1 	----MSPFLRCIARRRH~TTTATSVSILRPAATSRMLSTLPRLPLFEAVSKHDPDSKVVV
AN3417.1  	AYSTEYDDLSTFFWIEA1SFNCTSCPTFFGFAVRRSLATLPNLPLFRALKNHDPARLAVV
          	: ::              : ..:: .     *     :***.**:*.*: .*:*   .**

MG04646.1 	HSLSGRPFRYGGLLADVARVRDRLCEASGK--RELDGERVAFLVENSYDYVV1TLLAILA
NCU02107.1	HSNSGRTFKYGELLGDVCKTRNRLYETAGK--EDLDGERIAFLVENSYDYVV1TLLACLA
FG00287.1 	HSISGRTFKYGELLGDVSRARGRIAEAAGR--RDLNGERIAFLVENSYDYVV1TLLAAMA
AN3417.1  	HNPSFRSFTYGNLVADVVKAQDQLWNSSGRGRDGLRGERIAFLAENSYDYVV1ILLSILA
          	*. * *.* ** *:.** :.:.:: :::*:.   * ***:***.********  **: :*

MG04646.1 	AKSIAVPLAPAFPPPELQYILDHSEAALLLSSSKFAQKAQDVLATGLASKPTLLELSKFQ
NCU02107.1	AKSIAVPLSPAFPAPELQYILNHSEALLLLSSAKFTNKAQEVLKTELDSKPTLLELTKFQ
FG00287.1 	ARSIAVPLSPAFPAPELQYILNQSEASLLVSSPKFASKAKEVLATELNTKPAHLELQKHQ
AN3417.1  	SDAIAVPLSTGFPIHELKYIMNNSQAGMLVATERYADMAEKIMEGELDRQPILDMRGKIR
          	: :*****:..**  **:**:::*:* :*::: :::. *:.::   *  :*      * :

MG04646.1 	GDSAAPSEPITLDGGS-PGNAGMMLYTSGTTNRP0K--------G~VLLPQDVMTAQARS
NCU02107.1	G--GSQHEQVTLDSKSGPGEAGMMLYTSGTTNRP0K--------G~VLIPQSVMTAQARS
FG00287.1 	G--GGNHEKVELGDAD-PGEAGMMLYTSGTTNKP0K--------G~VLLPQAVLTAQSRS
AN3417.1  	TG-SSDVGAVELEGLD-GNSGGMMLYTSGTTNRP~VRLLADLADG1VVIPQSALASQAAS
          	 . ..    : * . .  ...***********:*     :. :.* *::** .:::*: *

MG04646.1 	LLQAWEYSPKDHLLHVLPLHHIHGTINALFAPLMAGSTIEFLFPFNADAVWRRLASPFMA
NCU02107.1	LIHAWEYTPADHLLHVLPLHHIHGTINAIFTPLFAGSSIEFMFPFNPDAVWKRFAAPFLP
FG00287.1 	LTEAWNYSPSDHLLHVLPLHHIHGTVNAILTPLLAGSTIEFMFPFNADAVWKRFSSPFLP
AN3417.1  	LLEAWKYSPGDRLLHLLPLHHIHGTVNAIITPVLSGSSIEFMFPFNPTAVWNRLAEPFLP
          	* .**:*:* *:***:*********:**:::*:::**:***:****. ***.*:: **:.

MG04646.1 	DPEASTNGSNGSAAPKKAPVTFFTVVPTVYSRLLATHKSLPADVLTAAREAVSPKNMRLS
NCU02107.1	DTSD------------KKKITFFTVVPTVYSRLLTTHKNLPGDLQTATRKAIAPSNLRLA
FG00287.1 	NDASIP----------KEKITFFTVVPTVYSKLLSSHKSLPPDMQEASRKAISPENMRLS
AN3417.1  	EGTK-------------DKITFLNAVPTIYNRLLSTFPTLSPQVQSASKVAISPEHLRLN
          	:                  :**:..***:*.:**::. .*. ::  *:: *::*.::** 

MG04646.1 	ISGSAALPTPVKRAWKELSGGTVLLERYGMTEVGMALSCGLADADRVDGSVGWPLPSVEA
NCU02107.1	ISGSAALPTPIKKAWHELSSGNVLLERYGMTEVGMALSCGLETADRVDGSVGWPLPGVEA
FG00287.1 	ISGSAALPTPIKTAWKDLSHGNVLLERYGMTEVGMALSCGLDFSDRVDASVGWPLPSVEA
AN3417.1  	ISGSAALPTPTKKAWQDLSNGNVLLERYGMTEVGMALSCGLDFTDRVDGSVGWPLPSVEV
          	********** * **::** *.*******************  :****.*******.**.

MG04646.1 	RLVDVDSGEVIKPGEEVDAE-GRERAGEIQLRGPTIFAEYWRNPEATAKEFVEDDTATAN
NCU02107.1	RLVDIENNTIIEPGQERDPETGRERAGEIQLRGPSIFREYWRNPEATAKEFVTDEKDVAG
FG00287.1 	RLVDVDTNDVIKEGEEVDAQ-GRERSGEIQLRGPTIFREYWRNPSATASEFVEGE----D
AN3417.1  	RLVDTDTNKVIQPGEEIDGS-GRAREGEIQLRGPTIFREYWGNEKATQETFAPDA----D
          	**** :.. :*: *:* * . ** * ********:** *** * .** . *. .     .

MG04646.1 	GNGHAANGHGRTAWFKTGDVAVRRPVPA-AGQNPAPGQEWTHQGPMYFILGRKSADIIKS
NCU02107.1	SKGR---------WFKTGDVAVRRPAPEGAGKSDLKSQKDWARGDMYFILGRKSADIIKS
FG00287.1 	GQGE---------WFKTGDVAVRRPVPS------ANAAQSWNHGPLYFIQGRKSADIIKT
AN3417.1  	GKGP---------WFKTGDVATRRSVET----AGLGKSGEWAKGPMYFIQGRRSVDIIKV
          	.:*          ********.**..                :* :*** **:*.**** 

MG04646.1 	GGEKVSALEVERELLSL------------------DQVAEAAVCAVPSGKWGQKVGCVIV
NCU02107.1	GGEKVSALEVEREMLSL------------------PQVAECAVLAVPSGKWGQKVGAVVI
FG00287.1 	GGEKVSALEVERELLSL------------------PQVSEAAVVAVPSGQWGHKVGAVLV
AN3417.1  	GGEKVSALEVERELLSLYVHQVLPSSLQELIEVTRPQIAEAAVVGLPSEQWGQKVVAIVV
          	*************:***   .  .:: ..  . :  *::*.** .:** :**:** .:::

MG04646.1 	LDKEHCADG-----KWSPMDMRRALKGRLVNYKIPQVMRVVE-HIPRNAMGKINKKQLVA
NCU02107.1	LDKEHVPEG-----RWTPLEMRRALKDRLAAYKIPQVLKIVD-KIPRNAMGKINKKNLVK
FG00287.1 	LDTDVVK-------KWSALDMRRALKGRLASYKIPQVMRLVD-QIPRNAMGKINKKQLVK
AN3417.1  	LNSEVAARTGRNGKSWGVLDMRWALKDRLAAYKLPSEMKVLEGAIPRNAMGKG--TVLLL
          	*:.:     . ... *  ::** ***.**. **:*. :::::. ********   . *: 

MG04646.1 	AVFQEDFSGDEM
NCU02107.1	EVFADEFSGDEL
FG00287.1 	AIFPDDISGDES
AN3417.1  	TSFTSLVPC---
          	  * . ..
```
